# Challenges in Diagnosing Addison's Disease: A Case Report

**DOI:** 10.7759/cureus.70178

**Published:** 2024-09-25

**Authors:** Joana Nascimento, Frederico Silva, Tiago Vasconcelos, Inês G Simões, Raquel Pinho

**Affiliations:** 1 Internal Medicine, Centro Hospitalar Universitário do Algarve, Unidade de Portimão, Portimão, PRT

**Keywords:** acth, addison's disease, cutaneous hyperpigmentation, hormone replacement therapy, primary adrenal insufficiency

## Abstract

Addison's disease, or primary adrenal insufficiency, is a rare condition characterized by a deficiency of adrenocortical hormones due to the damage of the adrenal glands. This case report presents a 36-year-old woman with a history of intestinal obstruction caused by adhesions and bands, who visited the emergency department with postprandial vomiting and epigastric discomfort. On physical examination, notable findings included bronzed skin, cutaneous hyperpigmentation, hyperpigmented scars and tongue, orange-colored teeth, decreased muscle strength in all four limbs, and mild gait imbalance. The patient also exhibited hyponatremia and hyperkalemia, which are common features of this disease. The diagnosis was confirmed by a computed tomography scan, which revealed adrenal gland calcifications. The patient was treated with hydrocortisone and fludrocortisone, resulting in significant clinical improvement. This case report underscores the importance of recognizing this condition and considering it in patients with nonspecific symptoms (fatigue, weight loss, and gastrointestinal disturbances), enabling effective therapy, including hormone replacement and continuous monitoring, which is crucial to optimize prognosis and prevent future complications.

## Introduction

Addison's disease, also known as primary adrenal insufficiency, is a rare endocrine disorder characterized by the progressive destruction of the adrenal cortex, resulting in a significant deficiency in the production of glucocorticoid hormones (primarily cortisol) and mineralocorticoid hormones (primarily aldosterone) [1]. It has an estimated incidence of four cases per million people per year, with a prevalence of one case in 100,000 people, being more common in Europe and North America (one to four cases per 100,000), with an even distribution between sexes, although there is a slight predominance in females [2].

The etiology of Addison's disease is multifactorial, with autoimmunity being the primary cause in developed countries, accounting for about 70-90% of cases. Autoimmune adrenalitis is characterized by the presence of autoantibodies against steroidogenic enzymes, such as 21-hydroxylase [3,4]. Other relevant causes include adrenal tuberculosis, which remains an important cause in endemic regions, infections, adrenal hemorrhage, malignancy, and genetic causes, particularly in male patients [5-7].

## Case presentation

We present the case of a 36-year-old woman with a history of hypothyroidism (with negative thyroid peroxidase antibodies), factor V deficiency diagnosed in the context of deep vein thrombosis of the lower limb, and a recent intestinal obstruction due to adhesions and bands about a month ago. The postoperative period was complicated by septic shock, due to bacterial translocation, necessitating invasive mechanical ventilation and inotropic support. Her regular medications included warfarin, levothyroxine, Neurobion (cyanocobalamin + pyridoxine + thiamine), metoclopramide, and trazodone.

After discharge, the patient developed postprandial vomiting, associated with epigastric discomfort and significant weight loss (37.5% over six months). She was evaluated at the surgery clinic, where a CT scan showed no abnormalities, and metoclopramide 10 mg was prescribed in case of nausea and/or vomiting. Subsequently, she was prescribed escitalopram for suspected depressive syndrome, which led her to seek emergency care.

**Figure 1:**
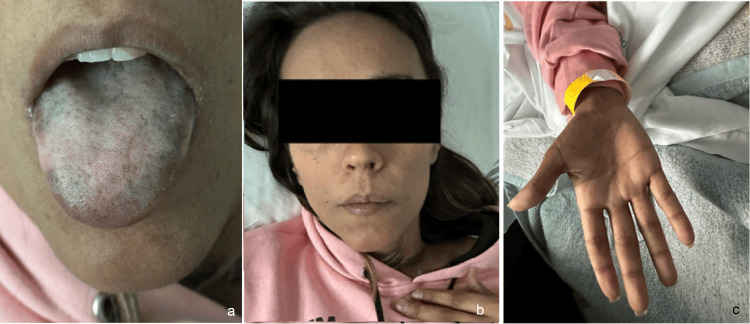
(a) Black-blue pigmentation of the lips; (b) Skin hyperpigmentation; (c) Skin hyperpigmentation

Due to persistent symptoms, she returned to the emergency department, where hypotension, skin and subcutaneous hyperpigmentation, a dark-toned tongue (Figure 1), orange-tinted teeth, generalized muscle weakness, and slight gait imbalance were noted.

The most relevant laboratory analysis (Table 1) revealed hyponatremia (sodium (Na) 130 mmol/L), hyperkalemia (potassium (K) 5.5 mmol/L), anemia (hemoglobin (Hb) 10.5 g/dL), and acute renal failure (creatinine 2.22 mg/dL). Suspecting adrenal insufficiency, adrenocorticotropic hormone (ACTH) and cortisol levels were measured, showing elevated ACTH (1408 pg/mL) and low cortisol (1.4 µg/dL).

**Table 1:** Relevant laboratory bloodwork findings on admission. Na+, sodium; Cl-, chloride; K+, potassium; ACTH, adrenocorticotropic hormone

Variable	Patient results	Normal values
Hemoglobin (g/dL)	10.5	13.8-17.2
Leucocyte (×10^9^/L)s	6600	4000-10000
Platelets (×10^9^/L)	174	150-400
C-reactive protein (mg/L)	2.1	<10
Total proteins (g/dL)	7.0	6.0-8.3
Albumin (g/dL)	4.0	3.4-5.4
Urea (mg/dL)	93	5-32
Creatine (mg/dL)	2.22	0.7-1.3
Na+ (mmol/L)	130	135-145
K+ (mmol/L)	5.5	3.6-5.2
Cl- (mmol/L)		96-106
ACTH (pg/mL)	1408	7.0 – 63.0
Cortisol (µg/dL)	1.4	6.2 – 19.4
Aldosterone (ng/dL)	4.6	3.7 - 31
Plasma renin (uU/ml)	345.0	4.2 – 59.7
Anti-adrenal antibody	Negative	

An abdominal and pelvic CT scan revealed bilateral adrenal calcifications, consistent with adrenal insufficiency. Treatment with hydrocortisone and fludrocortisone was initiated, leading to clinical and analytical improvement, with resolution of hyperpigmentation and gastrointestinal and renal symptoms.

## Discussion

Addison’s disease presents a diagnostic challenge due to the nonspecific nature of its early symptoms and the insidious progression of the condition [2]. This case report highlights several crucial aspects of diagnosing and managing Addison’s disease.

Symptoms of Addison’s disease are often nonspecific, which can delay diagnosis. Patients typically present with chronic fatigue, anorexia, weight loss, nausea and vomiting, and psychiatric disturbances. Skin and mucosal hyperpigmentation, resulting from elevated ACTH production by the pituitary in response to cortisol deficiency, is a hallmark clinical finding [8]. Orthostatic hypotension is also common, reflecting aldosterone deficiency, which leads to sodium loss and hypovolemia [9]. In advanced stages or during an adrenal crisis, patients may exhibit mental confusion, hypoglycemia, and severe hyponatremia, constituting a medical emergency [10].

In this case, the patient exhibited skin hyperpigmentation and nonspecific symptoms such as fatigue and weight loss, which are typical findings in Addison’s disease [3]. Hyperpigmentation results from increased ACTH production, which stimulates melanogenesis [4]. The initial evaluation also revealed persistent gastrointestinal disturbances, hypotension, and ionic abnormalities, including hyponatremia and hyperkalemia, common in adrenal insufficiency [5].

Diagnosis of Addison’s disease relies on a combination of clinical and laboratory findings. Laboratory confirmation includes low serum cortisol levels associated with elevated ACTH levels, suggesting primary adrenal insufficiency. The ACTH stimulation test assesses adrenal reserve, with a subnormal response confirming the diagnosis [11]. Additionally, hyponatremia and hyperkalemia are frequent laboratory findings reflecting aldosterone deficiency [12], as evidenced in this patient. Identification of specific antibodies, such as anti-21-hydroxylase antibodies, helps infer an autoimmune etiology for the disease [13]. The diagnosis was confirmed through imaging studies and laboratory analysis, revealing bilateral adrenal gland calcifications, elevated ACTH with decreased cortisol levels, and electrolyte disturbances such as hyponatremia and hyperkalemia [8,11].

Appropriate treatment for Addison’s disease involves hormonal replacement therapy, including glucocorticoids such as hydrocortisone, administered in doses that mimic physiological cortisol secretion, and mineralocorticoids like fludrocortisone to maintain electrolyte balance and blood pressure [14]. The patient was treated with hydrocortisone and fludrocortisone, resulting in significant symptom improvement and normalization of laboratory parameters [9]. Positive response to glucocorticoid treatment is an important indicator of diagnostic adequacy, as correcting hormonal deficiencies alleviates symptoms and improves quality of life [10].

This case also underscores the necessity of considering Addison’s disease in patients with nonspecific symptoms and a history of acute stress, such as postoperative complications. Primary adrenal insufficiency may manifest after stress-inducing events, such as septic shock, which can precipitate or exacerbate the condition [13]. Moreover, a comprehensive diagnostic approach, including clinical, laboratory, and imaging evaluations, is crucial for accurate diagnosis and effective management of Addison’s disease [14].

## Conclusions

The diagnosis of Addison’s disease is a significant challenge in clinical practice due to its variable presentation and overlap with other conditions. Cutaneous hyperpigmentation, combined with hyponatremia and/or hyperkalemia, provides a high index of suspicion. Treatment with glucocorticoids and mineralocorticoids is essential to control symptoms and prevent complications.

This case illustrates the importance of considering adrenal insufficiency in patients with nonspecific symptoms and highlights the effectiveness of glucocorticoid and mineralocorticoid therapy in improving symptoms and quality of life. Acute adrenal insufficiency is associated with high morbidity and mortality rates if not promptly recognized and treated. Therefore, increased awareness of Addison’s disease is crucial to improving patient outcomes and preventing serious complications, such as adrenal crisis and multiple organ dysfunction. With appropriate treatment, most patients can have a normal life expectancy and a good quality of life. However, it is crucial that patients are regularly monitored to adjust medication doses and avoid adrenal insufficiency secondary to stress or intercurrent illnesses.
